# Evaluation of Oral Nano-Silymarin Formulation Efficacy in the Prevention of Hand-Foot Syndrome and Neuropathy Induced by XELOX or m-FOLFOX6 Regimens in Metastatic Colorectal Cancer: A Triple-Blinded, Randomized Clinical Trial

**DOI:** 10.5812/ijpr-152364

**Published:** 2024-12-16

**Authors:** Hedyieh Karbasforooshan, Hossein Rahimi, Omid Arasteh, Abolghasem Allahyari, Mehdi Varmaghani, Mahdi Jannati, Vahid Ghavami, Mahmoodreza Jaafari, Sepideh Elyasi

**Affiliations:** 1 Department of Clinical Pharmacy, School of Pharmacy, Mashhad University of Medical Sciences, Mashhad, Iran; 2 Department of Internal Medicine, Ghaem Hospital, Mashhad University of Medical Sciences, Mashhad, Iran; 3 Department of Hematology-Oncology, School of Medicine, Mashhad University of Medical Sciences, Mashhad, Iran; 4 Department of Management Sciences and Health Economics, School of Health, Mashhad University of Medical Sciences, Mashhad, Iran; 5 Social Determinants of Health Research Center, Mashhad University of Medical Sciences, Mashhad, Iran; 6 Department of Epidemiology and Biostatistics, School of Health, Mashhad University of Medical Sciences, Mashhad, Iran; 7 Nanotechnology Research Center, Pharmaceutical Technology Institute, Mashhad University of Medical Sciences, Mashhad, Iran; 8 Department of Pharmaceutical Nanotechnology, School of Pharmacy, Mashhad University of Medical Sciences, Mashhad, Iran

**Keywords:** Nano-Silymarin, Metastatic Colorectal Cancer, XELOX, m-FOLFOX6, Hand-Foot Syndrome, Neuropathy

## Abstract

**Background:**

Folinic acid, fluorouracil, and oxaliplatin (FOLFOX) and oxaliplatin and capecitabine (XELOX) are the most widely used chemotherapy regimens for treating metastatic colorectal carcinoma (CRC). These regimens are associated with various adverse reactions, including neuropathy and hand-foot syndrome (HFS). Silymarin, a flavonoid derived from *Silybum marianum*, has a wide range of biological activities. It has been used to counteract chemotherapy side effects due to its antioxidant, anti-apoptotic, and anti-inflammatory properties.

**Objectives:**

The purpose of this study was to assess the preventive effect of nano-silymarin on neuropathy and HFS induced by the FOLFOX6 and XELOX regimens.

**Methods:**

A randomized, triple-blinded, placebo-controlled clinical trial was conducted on 60 patients who were randomly assigned to receive 70 mg capsules containing 15% silymarin nano micelles twice a day after meals, starting from the first day of the first chemotherapy course and continuing for six courses of the XELOX or m-FOLFOX6 regimen. The severity of adverse effects was assessed after the third and sixth courses based on the National Cancer Institute Common Terminology Criteria for Adverse Events (NCI-CTCAE) version 5.

**Results:**

The median CTCAE scores for HFS and neuropathy were significantly lower in the nano-silymarin group at the end of the third course (P < 0.001). However, the difference remained significant only for HFS at the end of the sixth course (P = 0.022). Additionally, the scores increased significantly in both the placebo and nano-silymarin groups during the therapy (P < 0.05).

**Conclusions:**

Nano-silymarin may be considered an adjuvant medication for the prevention of certain chemotherapy-induced adverse reactions. Further research with larger sample sizes and various doses of nano-silymarin is recommended for a more comprehensive evaluation.

## 1. Background

Colorectal cancer (CRC) is the third most frequently diagnosed cancer and the second leading cause of cancer-related fatalities (1). Up to 50% of patients with CRC develop metastasis (2), often involving the liver, which occurs in nearly half of CRC patients during the course of the disease (3). Metastatic colorectal cancer (mCRC) has a poor prognosis, with a 5-year survival rate of 14% (4). Treatment for these patients is palliative and generally consists of surgery, radiation therapy, and, most frequently, systemic chemotherapy (5). To improve patient survival, systemic chemotherapy has been the primary treatment modality for decades. Folinic acid, fluorouracil, and oxaliplatin and oxaliplatin and capecitabine (XELOX) are the most commonly used chemotherapy regimens for treating mCRC. Chemotherapy drugs can cause damage to various tissues and organs in the host (6). These complications hinder the quality of life of patients, potentially leading to treatment discontinuation or the use of sub-therapeutic doses. Additionally, these adverse effects, along with associated morbidity and mortality, significantly impact healthcare costs and impose a large burden on both the government and the population (7). Herbal medicines have been introduced to treat cancer and mitigate the side effects of chemotherapy due to their potential therapeutic mechanisms.

*Silybum marianum* L. (milk thistle) has been used as a medicinal plant for many years. It contains approximately 70 - 80% silymarin complex and around 20 - 30% chemically unspecified fractions, mainly composed of other polyphenolic compounds (8). Silymarin is a flavonolignan, and its water-alcoholic extract is derived from the seeds and fruits of the thistle plant. The pharmacological activities of silymarin are primarily attributed to silybin (9). Silymarin demonstrates anti-viral, immune-modulating, antioxidant, anti-inflammatory, and anti-proliferative effects (10, 11). Numerous in vitro and animal studies have reported that silymarin possesses a wide range of biological activities, such as fighting cancer (12-14) and managing chemotherapy-induced adverse reactions (12, 15). Silymarin exerts its anti-inflammatory effects by inhibiting the migration of neutrophils, reducing the production of leukotrienes, preventing the release of inflammatory mediators such as tumor necrosis factor (TNF)-α, interleukin (IL)-2, interferon (IFN)-γ, IL-1β, prostaglandin (PG) E2, PGF2, cyclooxygenase (COX)-2, and decreasing the production of nitric oxide. Silymarin also acts as an antioxidant by scavenging free radicals and inhibiting lipid peroxidation (16). 

In human and animal studies, silymarin has shown no specific side effects, and its side effects were comparable to those of a placebo. In rare cases, its use has been associated with gastrointestinal symptoms, headaches, confusion, and skin reactions. Silymarin has been shown to be safe for human consumption at therapeutic doses, even in doses of 700 mg three times daily for up to 24 weeks (17).

Numerous pre-clinical and clinical studies have examined the effectiveness of silymarin in preventing and treating complications caused by chemotherapeutic agents. However, there is a limited number of well-designed randomized clinical trials on this potential effect of silymarin, and further studies are crucial on this topic. Moreover, considering the low oral bioavailability of silymarin, the use of nano-silymarin in clinical studies may be beneficial. 

## 2. Objectives

The present study aimed to evaluate the effects of oral nano-silymarin on the management of hand-foot syndrome and neuropathy induced by XELOX or m-FOLFOX6 regimens, making it the first triple-blinded, randomized clinical trial of its kind. 

## 3. Methods 

### 3.1. Study Design 

This research was conducted as a triple-blind, balanced randomized, and placebo-controlled clinical trial in an oncology outpatient clinic in Mashhad, Iran, from January 2021 to July 2023. 

### 3.2. Study Population 

Patients with a diagnosis of mCRC who had Kirsten rat sarcoma virus (KRAS) and NRAS gene mutations, or who did not have these mutations but were not candidates for targeted therapy due to contraindications or financial incapacity, and who were treated with the XELOX or folinic acid, fluorouracil, and oxaliplatin (FOLFOX) chemotherapy regimen, were assessed for eligibility. The inclusion criteria for the study were as follows: Patients aged 18 - 70, adequate liver and kidney function based on the following parameters: Alanine transaminase (ALT), aspartate transaminase (AST) ≤ 5 x ULN, total bilirubin ≤ 2 x ULN, creatinine ≤ 1.5 mg/dL, daily performance status based on Eastern Cooperative Oncology Group (ECOG) criteria of 0 or 1, and written consent to enter the study. Patients were excluded if they were pregnant or lactating, had a history of allergy to silymarin, had multiple primary cancers, had a history of heart failure, hepatitis B or C, or had autoimmune disorders or acquired or drug-induced immunodeficiency (except those caused by chemotherapy). Additional exclusion criteria included unwillingness to continue the study, inability to swallow the capsule, participation in another similar study, receipt of other drugs that could affect the treatment response, worsening of the prescribed chemotherapy regimen, occurrence of intolerable side effects based on the National Cancer Institute Common Terminology Criteria for Adverse Reactions (NCI‐CTCAE) version 5 that required discontinuation of treatment, use of other antioxidant drugs, and being a candidate for curative surgery. 

### 3.3. Study Protocol 

The patients were initially assigned to either the nano-silymarin or placebo group in a 1:1 ratio by random selection. The trial involved using a dried extract of *S. marianum* as an intervention, which is approved for use as a natural health product in Iran and derived from the seeds of milk thistle. The treatment group received nano micelle capsules (70 mg) twice daily with meals (SinaLiveR; Exir Nano Pharmaceutical Company, Tehran, Iran, registered code: 6231566784211002) from the first day of the first chemotherapy course for six courses of the XELOX or modified 6-FOLFOX regimen. The placebo capsules were produced identically by the same manufacturer and contained all the components of the capsules, except for silymarin. 

Due to the lipophilic nature, limited water solubility (0.04 mg/mL) (18), fast liver processing, and inadequate absorption in the intestines, the bioavailability of silymarin is estimated to be between 20% and 50% (19). In this research, silymarin-containing nano micelle capsules were used to enhance oral bioavailability. Micelles are a novel drug delivery system in which drugs are physically entrapped within the hydrophobic region of the nano micelles (20, 21).

The modified 6-FOLFOX regimen includes a 2-hour infusion of oxaliplatin (85 mg/m²), leucovorin (400 mg/m²), and a bolus of 5-FU (400 mg/m²) on the first day, followed by a 5-FU infusion of 2400 mg/m² over 46-48 hours, repeated every two weeks (22). The XELOX regimen consists of an infusion of oxaliplatin (130 mg/m²) on day 1, followed by oral capecitabine (1000 mg/m²) twice daily from the evening of day 1 to the morning of day 15, with a 7-day treatment-free interval in a 3-week cycle (23).

### 3.4. Outcomes 

A list of possible side effects (neuropathy caused by oxaliplatin and hand-foot syndrome (HFS) caused by capecitabine) was prepared. At the end of each course, the patient was examined by the oncologist based on the CTCAE version 5 criteria (Appendix 1 in Supplementary File). The National Cancer Institute (NCI) of the National Institutes of Health (NIH) has published standardized definitions for adverse events (AEs), known as the CTCAE (also called "common toxicity criteria" [CTC]), to describe the severity of organ toxicity in patients receiving cancer therapy; causality is not required. The CTCAE is periodically updated by the NCI, and CTCAE version 5.0 (v5.0) was published in November 2017 and became effective in April 2018. In general, toxicity is graded as mild (grade I), moderate (grade II), severe (grade III), or life-threatening (grade IV), with specific parameters according to the organ system involved. Death (grade V) is used for some criteria to represent a fatality occurring during treatment (24).

During the study, the patients were monitored for their compliance with the prescribed treatment and any adverse reactions (ADR) to the medication. Adherence to the treatment was determined based on whether the patients consumed more than 80% of the recommended capsules. 

The patients were asked to self-report any adverse reactions during the study. Additionally, they were questioned about this by the clinicians at the time of chemotherapy. Since silymarin's side effects were comparable to the placebo in previous studies and, in rare cases, its use has only been linked to gastrointestinal symptoms, headaches, confusion, and skin reactions (17), we did not perform any laboratory or paraclinical evaluations to detect its ADR. 

### 3.5. Sample Size 

To the best of our knowledge, this study is the first clinical trial in this field, and we proposed it as a preliminary study. In line with Whitehead et al. recommendations, a pilot trial with 75, 25, 15, and 10 participants per treatment group is sufficient for standardized effect sizes of extra small (≤ 0.1), small (0.2), medium (0.5), or large (0.8), respectively (25), for a main trial with 90% power and a two-sided 5% significance level. Hence, the standardized effect size for nano-silymarin in this study is expected to be small to medium. Considering a study power of 90% (β = 0.20) and an α error of 5%, a sample size of 15 - 25 per group would be sufficient. Therefore, the sample size in each arm was determined to be 25 patients. Taking into account potential dropouts, 5 additional participants were added to each arm, resulting in a total of 30 patients selected for each study arm.

### 3.6. Randomization and Blinding

Randomization was performed based on a computer-generated list of random allocation sequences from the randomization.com site. Subsequently, block randomization with blocks of four patients was used to ensure a balanced allocation of eligible patients between the control and intervention arms. To maintain the study's concealment, both nano-silymarin and placebo soft gel capsules were placed in identical boxes and assigned a number between 1 and 60 based on the allocation sequence (by Exir Nano Sina Company), with silymarin and placebo labeled as A and B, respectively. This coding remained secret to the researchers until the analysis was completed, and the boxes were delivered to the clinical pharmacist. 

The medications were provided to the patients in two boxes: One at the beginning of the study and another after four weeks to better assess their compliance. Each box contained 120 soft gels (each containing 70 mg) of silymarin nano-micelles or placebo, sufficient for an eight-week supply. Patients who met the inclusion criteria were selected by an oncologist and provided with the boxes according to the allocation list. Both the clinical pharmacist and oncologist evaluated the patients during treatment without knowing which group the patients were assigned to. The person analyzing the data also remained unaware of the group allocation until the end of the study when the company decoded A and B.

### 3.7. Statistical Methods 

Data analysis was conducted using SPSS software (version 27.0). Data are presented as mean ± standard deviation or median (range) for continuous or discrete quantitative variables, respectively, and as count (percentages) for nominal variables. The normality of the variable distributions was assessed using the Kolmogorov–Smirnov test. Comparisons between the two groups were made using an independent sample *t*-test for quantitative variables and Fisher's exact test for qualitative variables. The Mann-Whitney test was used for quantitative data with non-normal distribution. A significance level of P < 0.05 was considered for all tests. 

## 4. Results 

### 4.1. Baseline Characteristics 

**Figure 1.**
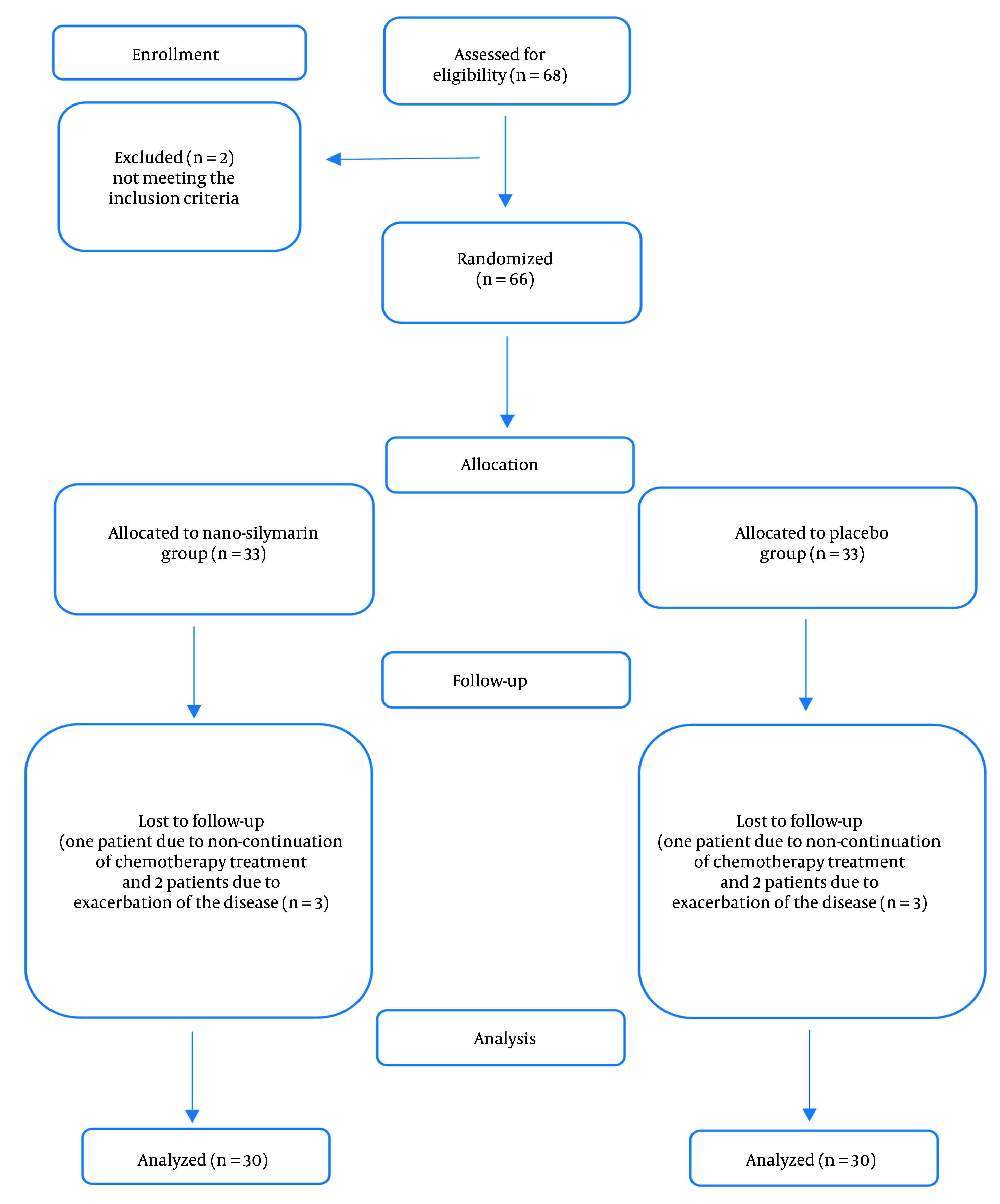
Flow diagram of the research design

A total of 66 subjects were eligible to participate in the study based on the inclusion criteria, out of 68 patients screened. Of the 33 patients included in each group, three dropped out from each group. Therefore, 30 patients in each group completed the study (Figure 1). The average age of the patients was 50.3 ± 11.17 years, with 50% of the patients being women. Seventy percent of the patients were receiving the FOLFOX regimen, while the remaining 30% were undergoing the XELOX regimen. The overall incidence of liver metastases was 30%. Table 1 summarizes the primary characteristics and biochemical parameters of the patients in both groups. All patients adhered to the nano-silymarin or placebo capsules during the 4-month follow-up. Apart from the location of metastasis, there were no significant differences in characteristics between the two groups.

**Table 1.** Patient Characteristics and Biochemical Parameters ^a^

Parameter	Nano-Silymarin Group (n = 30)	Placebo Group (n = 30)	P-Value
**Weight (kg)**	64.86 ± 10.51	68.43 ± 11.70	0.219 ^b^
**Height (cm)**	164.46 ± 10.51	168 ± 8.42	0.066 ^b^
**Body surface area (m ^**2**^ **)****	1.75 (1.59 - 1.81)	1.78 (1.65 - 1.94)	0.141 ^c^
**Gender **			0.606 ^d^
Female	14 (46.7)	16 (53.3)	
Male	16 (53.3)	14 (46.7)	
**Comorbidity disease**			
Diabetes	11 (36.7)	5 (16.7)	0.08 ^d^
Hypertension	5 (16.7)	6 (20)	0.739 ^d^
Cardiovascular diseases	0 (0)	1 (3.3)	1 ^d^
Hypothyroidism	0 (0)	1 (3.3)	1 ^d^
Hyperlipidemia	0 (0)	1 (3.3)	1 ^d^
**Concurrent medicines**			
Antidiabetic	11 (36.7)	5 (16.7)	0.08 ^d^
Antihypertensive	5 (16.7)	6 (20)	0.739 ^d^
Heart medications	0 (0)	1 (3.3)	1 ^d^
Hypothyroidism medications	0 (0)	1 (3.3)	1 ^d^
Lipid-lowering medications	0 (0)	1 (3.3)	1 ^d^
**ECOG**			1 ^d^
Zero	16 (53.3)	16 (53.3)	
One	14 (46.7)	14 (46.7)	
**Chemotherapy regimen**			0.091 ^d^
FOLFOX	24 (80)	18 (60)	
XELOX	6 (20)	12 (40)	
**Metastasis site **			0.009 ^d^
Liver	20 (66.7)	8 (26.7)	
Lung	0 (0)	5 (16.7)	
Peritoneum	8 (26.7)	8 (26.7)	
Lymph	0 (0)	3 (10)	
Liver and lung	2 (6.7)	5 (16.7)	
Liver and bone	0 (0)	1 (3.3)	
**Liver metastasis **			0.035 ^d^
Yes	22 (73.33)	14 (46.66)	
No	8 (26.66)	16 (53.33)	
**Number of metastatic site**	1 (1 - 1)	1 (1 - 1)	0.132 ^c^

Abbreviations: FOLFOX, folinic acid, fluorouracil, and oxaliplatin; ECOG, Eastern Cooperative Oncology Group; XELOX, oxaliplatin and capecitabine.

^a^ Values are expressed as mean ± SD or median (range) or No. (%).

^b^ Independent‐sample *t*-test.

^c^ Mann-Whitney test.

^d^ Chi‐squared test.

### 4.2. Efficacy of Nano-Silymarin on Chemotherapy-Induced Sensory Neuropathy 

After three courses of chemotherapy, the neuropathy grade remained at zero in 90% of patients in the treatment group. In contrast, in the placebo group, 63.3% of patients experienced grade I neuropathy, and six patients had grade II or III neuropathy. However, after the sixth course, most patients in both groups experienced neurotoxicity of grade I (56.7% vs. 40% in the placebo and silymarin groups, respectively). No patients in either group displayed neuropathy of grade IV or V (Table 2). 

**Table 2.** Frequency of Neuropathy and Hand-Foot Syndrome in the Two Groups After 3 and 6 Courses Based on National Cancer Institute Common Terminology Criteria for Adverse Events v.5 ^a^

Adverse Reaction and Group	Grade 0	Grade I	Grade II	Grade III	Grade IV	Grade V
**Neuropathy**						
Course 3						
Silymarin	27 (90)	3 (10)	0 (0)	0 (0)	0 (0)	0 (0)
Placebo	5 (16.7)	19 (63.3)	5 (16.7)	1 (3.3)	0 (0)	0 (0)
Course 6						
Silymarin	9 (30)	12 (40)	8 (26.7)	1 (3.3)	0 (0)	0 (0)
Placebo	5 (16.7)	17 (56.7)	7 (23.3)	1 (3.3)	0 (0)	0 (0)
**HFS**						
Course 3						
Silymarin	27 (90)	3 (10)	0 (0)	0 (0)	0 (0)	0 (0)
Placebo	10 (33.3)	18 (60)	1 (3.3)	1 (3.3)	0 (0)	0 (0)
Course 6						
Silymarin	21 (70)	4 (13.3)	3 (10)	2 (6.7)	0 (0)	0 (0)
Placebo	10 (33.3)	13 (43.3)	6 (20)	1 (3.3)	0 (0)	0 (0)

Abbreviation: HFS, hand-foot syndrome.

^a^ Values are expressed as No. (%).

The silymarin group showed a significantly lower CTCAE neuropathy score at the end of the third course (P < 0.05), but this difference was no longer observed at the end of the sixth course (Table 3). Additionally, the scores significantly increased in both groups during the six courses of treatment (P ≤ 0.001). However, the rate of increase was slower in the treatment group, as there was no significant difference between the baseline and third week scores in the treatment group (P = 0.083), in contrast to the placebo group (P < 0.05).

**Table 3.** National Cancer Institute Common Terminology Criteria for Adverse Events Chemotherapy-Induced Adverse Effects Scores for the Silymarin and Placebo Groups ^a^

Variables	Nano-Silymarin Group (n = 30)	Placebo Group (n = 30)	P-Value ^b^
**Neuropathy, at the beginning of the study**	0 (0 - 0)	0 (0 - 0)	1
**Neuropathy, at the end of course 3**	0 (0-0)	1 (1 - 1)	< 0.001 ^c^
**Neuropathy, at the end of course 6**	1 (0 - 2)	1 (1 - 2)	0.610
**HFS, at the beginning of the study**	0 (0 - 0)	0 (0 - 0)	1
**HFS, at the end of course 3**	0 (0 - 0)	1 (0 - 1)	< 0.001 ^c^
**HFS, at the end of course 6**	1 (0 - 1)	1 (0 - 1.25)	0.022 ^c^

Abbreviations: NCI-CTCAE, National Cancer Institute-Common Terminology Criteria for Adverse Events; HFS, hand-foot syndrome.

^a^ Values are expressed as median (quartile range).

^b^ Mann–Whitney test.

^c^ P < 0.05.

### 4.3. Efficacy of Nano-Silymarin on Chemotherapy-Induced Hand-Foot Syndrome

After three courses of chemotherapy, 90% of patients in the treatment group still had a zero HFS score. In contrast, in the placebo group, 60% had grade I HFS, and two patients showed higher grades. At the end of the sixth course, 70% of patients in the silymarin group still had no HFS, while 43.3% and 20% of patients in the placebo group experienced grade I and II HFS, respectively. No patients in either group displayed HFS of grade IV or V (Table 2). 

The CTCAE score for HFS was significantly lower in the silymarin group compared to the placebo group after both the third and sixth courses of therapy (P < 0.05) (Table 3). 

### 4.4. Safety of Treatment 

No adverse reactions related to silymarin or placebo capsules were observed among the patients. However, it should be noted that all patients received anti-emetics alongside their chemotherapy regimen, which could mask nausea. Additionally, 6.7% of patients in the treatment group experienced diarrhea after 3 and 6 courses of chemotherapy. This occurred in 63.3% of patients in the placebo group, and it is likely related to the chemotherapeutic agents. 

## 5. Discussion 

In this clinical trial, the efficacy of an oral formulation of nano-silymarin was evaluated for the prevention of HFS and neuropathy induced by two chemotherapy regimens, XELOX and m-FOLFOX6. Based on NCI-CTCAE scores, the severity of HFS was significantly lower in the silymarin group after both 3 and 6 courses, while neuropathy was significantly reduced only after course 3. 

In recent years, numerous in vitro and animal studies have reported a wide range of biological activities of silymarin, the main compound of *Silybum marianum*, including antioxidant, anti-apoptotic, and anti-inflammatory effects in preventing and treating complications caused by chemotherapeutic agents (16). 

Hand-foot syndrome is a dose-limiting side effect of capecitabine, occurring in 53 - 77% of patients (26). In many cases, this results in a reduction in the duration or intensity of cancer treatment. The pathogenesis of HFS is not completely understood, but a direct toxic effect on the palms and soles is considered the most likely cause. Capecitabine and its metabolites accumulate in these areas due to the increased levels of thymidine phosphorylase enzyme in the keratinocytes and the high concentration of the eccrine system, which eliminates capecitabine. Consequently, the thick stratum corneum of the palms and soles acts as a reservoir, leading to the production of toxic free radicals and oxidative stress (27).

Moreover, the COX inflammatory response can contribute to the pathogenesis of HFS. Chemokines mediating this reaction include IL-8, IL-1β, IL-1α, and IL-6 (28). A previous clinical study demonstrated that the severity of capecitabine-induced HFS can be significantly reduced by the prophylactic administration of a topical formulation of silymarin. However, to date, no in vitro or in vivo studies have investigated the efficacy of oral silymarin in the prevention of HFS. 

In a randomized, double-blind clinical trial conducted by Elyasi et al. in 2017, 40 patients were randomly assigned to receive either a 1% topical formulation of silymarin or a placebo twice daily on the palms of the hands and soles of the feet, alongside daily chemotherapy with capecitabine, continuing for 9 weeks. This study indicated that the use of a silymarin topical formulation for 9 weeks significantly reduced the severity of HFS caused by capecitabine and delayed its occurrence (29). 

Our research is the first clinical trial to assess the impact of oral nano-silymarin in preventing HFS in a triple-blinded, randomized fashion. In line with the study conducted by Elyasi et al., a notable difference was observed between the two groups in terms of the CTCAE HFS score after 3 and 6 treatment cycles. In contrast to the placebo group, none of the patients in the silymarin group had a grade higher than 1 after completing three courses of treatment. However, in Elyasi et al.'s study, the World Health Organization (WHO) HFS grading scale was used (29), which is one of the two most commonly used HFS classifications, alongside the NCI-CTCAE scale, for decisions regarding dose reduction or drug discontinuation (30). In their study, the scores increased significantly in both the placebo and silymarin groups during chemotherapy, but there was a delay in the development and progression of HFS in the silymarin group, which is exactly consistent with our findings.

Approximately 40% - 50% of patients who receive oxaliplatin experience dose-limiting peripheral sensory neuropathy (31). Acute neuropathy results from oxaliplatin infusion, while chronic neuropathy is a consequence of repeated dosing at cumulative doses exceeding 1000 mg/m². Oxaliplatin-induced degenerative damage in nerve cells may be attributed to apoptosis mediated by p38-mitogen-activated protein kinase (MAPK) and caspase-3, as well as inhibition of brain-derived neurotrophic factor (BDNF) expression. Oxaliplatin significantly enhances oxidative stress through lipid peroxidation and DNA and protein oxidation (32). Antioxidant compounds are a potential treatment option for this adverse effect (15). 

There is evidence supporting the neuroprotective effects of silymarin in neurodegenerative diseases, including Alzheimer's disease, Parkinson's disease, and cerebral ischemia (33). This may be due to the reduction of reactive oxygen species (ROS) and inflammatory cytokines, as well as the induction of the cell apoptosis pathway. Silymarin has been shown to increase BDNF expression while inhibiting ROS production. Additionally, silymarin protects cells from the activation of caspase-3 apoptotic signaling induced by oxaliplatin (32). Two in vitro studies demonstrated that silymarin administration induced beneficial effects in oxaliplatin-induced neuropathy through the inhibition of oxidative stress and apoptosis (32, 34). In an animal study, silymarin (100 mg/kg for 20 days) was shown to have protective effects against neuropathy, attributed to its anti-apoptotic and antioxidant properties (35). 

However, no clinical trials have yet assessed the efficacy of silymarin in the prevention of oxaliplatin-associated neuropathy. In a study involving patients with neuropathy following chemotherapy regimens containing platinum compounds, taxanes, and vinca alkaloids, the administration of silymarin at a dose of 140 mg twice daily for 3 months led to a notable improvement in neuropathy symptoms (36). In the present study, oral nano-silymarin— which has higher bioavailability—at a lower dose and for a longer period (70 mg twice a day for 4 months) effectively prevented neuropathy up to the completion of the third treatment cycle. In fact, nano-silymarin could not maintain its effect until the end of the sixth course and was only able to delay the occurrence of complications. However, in contrast to the placebo group, no participant in the silymarin group experienced neuropathy grades higher than 1 during the study. Despite this, neuropathy levels increased over time in both groups during the six courses of chemotherapy.

The use of a nano-formulation was a key advantage of our study. The nanomicelles are approximately 10 nm in size and provide nearly 100% encapsulation of silymarin. This significantly enhanced the solubility of silymarin in water by 3,000 times, thus protecting it from the destructive effects of gastric fluids. Furthermore, the nanomicelles remain intact in the acidic environment of the stomach for at least three hours and retain their original characteristics upon reaching the small intestine. Nanomicelles also facilitate the transport of silymarin across the epithelial cell layer in the intestine, leading to improved absorption (37). An in vitro study demonstrated that nano-formulation increases the bioavailability of silymarin (38). In another study, the absorption of silymarin micelles in various segments of the intestine was significantly higher than that of free silymarin in rats (21). Additionally, an animal study in rats showed that administration of nano-silymarin at a dose of 5 mg/kg for 14 days provided protection against 5-FU-induced gastrointestinal toxicity (39).

However, the study had certain limitations. First, the sample size was limited. Since there were no prior human studies on the use of silymarin to prevent these two ADRs, we considered this study a pilot study for sample size calculation. Future studies based on our findings could increase the power by using a larger sample size. Additionally, further research is needed to explore the effectiveness and safety of various doses of nano-silymarin in a larger population and over an extended duration. 

Second, we only assessed sensory neuropathy, and motor neuropathy was not evaluated. Additionally, we did not perform electromyography (EMG) or nerve conduction velocity (NCV) tests. Hand-foot syndrome could also be assessed using other tools, such as the WHO scale, but we only used the NCI-CTCAE scale. 

Third, we did not compare the efficacy of nano-silymarin with conventional formulations, which could be a suggestion for future research. 

Fourth, since all the included patients received antiemetic drugs (aprepitant and ondansetron) for the management of chemotherapy-induced nausea and vomiting, we could not evaluate one of the most commonly reported adverse reactions of silymarin in previous studies—nausea and vomiting. None of the patients in our study reported this complaint. 

Fifth, we did not assess any markers (e.g., serum levels of inflammatory mediators like TNF-α or IL-6) that could help predict the probable mechanism of action of silymarin. Future studies could investigate the mechanism of action of silymarin and explore the use of other antioxidants in combination with silymarin (e.g., vitamin C or N-acetylcysteine), which was not addressed in our trial. 

Finally, we did not report the potential effects of oral silymarin administration on chemotherapy efficacy, particularly given recent promising data on silymarin’s use as an adjuvant to chemotherapy in various cancers. This may be better assessed in future studies. We followed up with patients in this regard, and the data will be published in the near future.

### 5.1. Conclusions 

This study demonstrated that an oral nano-formulation of silymarin, at a daily dose of 140 mg divided into two equal doses for 6 courses of chemotherapy alongside the XELOX or m-FOLFOX6 regimen, may significantly prevent hand-foot syndrome and at least delay the onset of neuropathy in patients with mCRC. Further clinical trials with larger sample sizes and varying doses, as well as the use of more advanced assessment tools such as EMG-NCV for neurotoxicity, are recommended. 

ijpr-23-1-152364-s001.pdf

## Data Availability

The data supporting the findings of this study are available from the corresponding author upon reasonable request.

## Clinical Trial Registration Code:

IRCT20200408046990N5.
