# Supplementary material for: Evaluation of Oral Nano-Silymarin Formulation Efficacy in the Prevention of Hand-Foot Syndrome and Neuropathy Induced by XELOX or m-FOLFOX6 Regimens in Metastatic Colorectal Cancer: A Triple-Blinded, Randomized Clinical Trial
Source: Iran J Pharm Res. 2024 Dec 16;23(1):e152364. doi: 10.5812/ijpr-152364 (PMC11892790; doi:10.5812/ijpr-152364)
Supplement: ijpr-23-1-152364-s001.pdf [file ijpr-23-1-152364-s001.pdf]

Appendix 1: National Cancer Institute Common Terminology for Adverse Events (NCI-CTCAE) scoring system version 5 (21)

CTCAE term	Grade 1	Grade 2	Grade 3	Grade 4	Grade 5
**Peripheral sensory neuropathy**	Asymptomatic	Moderate symptoms; limiting instrumental ADL	Severe symptoms; limiting self-care ADL	Life-threatening consequences; urgent intervention indicated	Death
**Hand-foot syndrome**	Minimal skin changes or dermatitis (e.g., erythema, edema, or hyperkeratosis) without pain	Skin changes (e.g., peeling, blisters, bleeding, fissures, edema, or hyperkeratosis) with pain; limiting instrumental ADL	Severe skin changes (e.g., peeling, blisters, bleeding, fissures, edema, or hyperkeratosis) with pain; limiting self-care ADL	-	-
